# Frequency of Non-motor Symptoms in Parkinson's Patients With Motor Fluctuations

**DOI:** 10.3389/fneur.2021.678373

**Published:** 2021-06-29

**Authors:** Mariana Fernandes, Mariangela Pierantozzi, Alessandro Stefani, Carlo Cattaneo, Erminio A. Bonizzoni, Rocco Cerroni, Nicola Biagio Mercuri, Claudio Liguori

**Affiliations:** ^1^Department of Systems Medicine, Sleep Medicine Center, University of Rome “Tor Vergata,” Rome, Italy; ^2^Neurology Unit, University Hospital of Rome Tor Vergata, Rome, Italy; ^3^Parkinson's Disease Center, University Hospital of Rome Tor Vergata, Rome, Italy; ^4^Medical Department, Zambon SpA, Bresso, Italy; ^5^Department of Clinical Science and Community, Section of Medical Statistics and Biometry “GA Maccacaro,” University of Milan, Milan, Italy; ^6^Scientific Institute for Research, Hospitalization and Healthcare (IRCCS) Fondazione Santa Lucia, Rome, Italy

**Keywords:** idiopathic Parkinson disease, motor fluctuations, sleep, psychiatric disorder, fatigue, real-word data

## Abstract

**Background:** Non-motor symptoms (NMS), including neuropsychiatric, sleep, autonomic, and sensory domains, are an integral aspect of the clinical presentation of Parkinson disease (PD) and affect neurocognitive functioning as well as patients' and caregivers' well-being.

**Objective:** To describe the occurrence of NMS in PD patients with motor fluctuations in real-life condition.

**Methods:** The present study is a secondary analysis of a previous multinational, multicenter, retrospective-prospective cohort observational study (SYNAPSES). Patients with PD diagnosis and motor fluctuations aged ≥18 years were included. Data collected at the baseline visit were used for this study, and descriptive analyzes were conducted to describe the distribution of NMS in motor-fluctuating PD patients distributed according to different clinical characteristics.

**Results:** Of the 1,610 patients enrolled, 1,589 were included for the analysis (978 males and 611 females), with a mean age of 68.4 (SD = 9.6). Most patients had at least one NMS (88.5%). Sleep problems and psychiatric symptoms were the most prevalent NMS in motor fluctuating PD patients in all H and Y stages. Psychiatric disorders were more frequent in older patients and in patients with a larger number of years of PD diagnosis, while sleep problems were more preeminent in younger patients and with inferior disease duration.

**Conclusions:** The present findings further support the high prevalence of NMS in PD patients with motor fluctuations, thus reinforcing the need for assessing them for diagnostic accuracy and for delivering holistic care.

## Introduction

The occurrence of dyskinesias and motor fluctuations, such as wearing off and on-off effects, is a crucial problem in the long-term management of patients with Parkinson's disease (PD) (1, 2). Motor fluctuations are related to a variety of factors, including the age of disease onset, disease duration, duration of treatment, disease severity, and dose of levodopa (3–8). Moreover, it has been documented that advanced PD patients with motor fluctuations tend to report non-motor symptoms (NMS) more frequently than mild-moderate patients, and the NMS themselves tend to be more severe and sometimes also fluctuating (9).

The recognition and treatment of NMS have increasingly become the focus of clinical practice and research in the recent past. In particular, from the first description of NMS, it has been documented that a large proportion of PD patients may show neuropsychiatric disturbances, sleep disorders, fatigue, gastrointestinal symptoms, autonomic dysfunction, and sensory complaints (10–13). Moreover, NMS has been documented to be present from the prodromal or early stages of the disease and vary along the disease duration (14).

NMS has been shown to have a strong impact on PD patients' functioning and daily living since they significantly affect daytime activities and impair patients' and caregivers' well-being (10, 12, 13). The main common NMS in PD are depression, anxiety, apathy, sleep disorders, pain, fatigue, autonomic dysfunction, and gastrointestinal disturbances, which can be present in both early and moderate-advanced stages of the disease (10, 12, 13, 15, 16). In contrast to the evident motor dysfunction in PD patients, NMS remain often under-recognized in clinical practice, although they are highly common (17, 18). Consistently, a survey has reported that nearly 62% of NMS in PD may remain undeclared to healthcare professionals since patients may be unaware that the symptoms are associated with PD (17).

Motor fluctuations (either early or advanced) can significantly add to the NMS burden (9, 19–21). However, few studies specifically focused on the NMS prevalence in motor-fluctuating PD patients, who usually need higher support from clinicians and caregivers (20, 22). Therefore, the main aim of the present study, obtained from a real-world experience, was primarily to describe the prevalence of clinically recognized NMS in motor-fluctuating PD patients and to characterize their occurrence both according to the staging of the disease and the cardinal motor symptoms and finally to evaluate a possibly gender-based difference in their manifestation.

## Methods and Materials

### Study Design

The present study is a secondary analysis of a previous multinational, multicenter, retrospective-prospective cohort observational study (SYNAPSES; EU PAS Register Number EUPAS - 13745). For this descriptive study, we specifically included data obtained from the baseline visit in which patients complained of NMS after being carefully examined by clinicians. Globally, the SYNAPSES study was conducted in 128 neurology and geriatric centers, specialized in PD treatment, across several countries, namely Belgium, Germany, Italy, Spain, Switzerland, and the United Kingdom. The main aim of the SYNAPSES study was to monitor the effectiveness and tolerability of safinamide in motor fluctuating PD patients. All patients reporting motor fluctuations were enrolled and then observed at a 12-month follow-up. The original protocol and the main study results were published in Abbruzzese et al. (23).

The trial was approved by Independent Ethics Committees and Health Authorities in all the countries involved in the study and was conducted according to the ethical standards of the institutional and/or national research committee and the Declaration of Helsinki.

### Participants

There were 1,610 PD patients enrolled in this study. Male and female patients with motor fluctuations aged ≥18 years who had started treatment with safinamide at the enrollment visit or in the previous 4 months, according to clinical practice, and who had signed informed and privacy consent forms were included in the study. Inclusion and exclusion criteria and characteristics of patients have been reported previously (23).

### NMS Collection

NMS data were collected at baseline and recorded in electronic standardized case report forms (eCRFs). For all patients enrolled in the study, NMS were recorded by the clinicians by selecting items from a menu already available in the eCRF. The NMS listed on the menu had previously been selected from the main NMS symptoms described in the literature and had the approval of the Steering Committee members. In order to better identify the NMS burden, all clinicians checked the NMS impact and gravity from the medical charts and patient interviews. Then, following the categories described in the literature, NMS were labeled as attention disorders, cardiovascular symptoms, cognitive symptoms, fatigue, pain, respiratory symptoms, gastrointestinal symptoms, urinary symptoms, primary sensory symptoms, psychiatric symptoms, skin disorders, sleep disorders, and other non-motor symptoms.

### Statistical Analyzes

Because of the observational and descriptive nature of the SYNAPSES study, the aims of the study were addressed using descriptive statistics. Descriptive statistics have been provided by tabulating absolute and relative frequency through two-way contingency tables. The relative frequencies were calculated on patients with missing and non-missing data. Stratifications by country were not predicted because no differences among countries were detected. Computations were performed using SAS® version 9.4.

## Results

From the initial 1,610 patients enrolled in the study, 1,589 (98.7%) were considered for this analysis. Males represent the majority of the sample (61.55%, *n* = 978), whereas females accounted for 38.45% (*n* = 611). The mean age of the entire sample was 68.41 (SD = 9.63) and the mean years from diagnosis was 7.94 (SD = 5.40). Out of this final sample, 1,406 patients (88.5%) reported at least one NMS. As shown in Table 1, considering the whole sample of patients, we found that sleep disorders and psychiatric symptoms were the most prevalent complaints, followed by fatigue, gastrointestinal symptoms, urinary symptoms, pain, and cognitive symptoms. In view of the gender-based analysis, psychiatric problems were highly frequent among women, whereas sleep problems were the most common NMS reported among men (see Table 2).

**Table 1:** Distribution of non-motor symptoms in idiopathic Parkinson's disease.

	**% (*n*)**
Males, % (*n*)	61.55 (978)
Mean age (SD)	68.41 (9.63)
Mean years from diagnosis (SD)	7.94 (5.40)
Patients with at least one NMS, % (*n*)	88.5 (1,406)
**Non-motor symptoms (*****n*** **=** **1,589)**	
Attention disorders	12.8 (204)
Cardiovascular symptoms	9.4 (150)
Cognitive symptoms	16.7 (266)
Fatigue	25.5 (405)
Gastrointestinal symptoms	23.7 (376)
Pain	20.9 (332)
Primary sensory symptoms	7.9 (125)
Psychiatric symptoms	43 (684)
Respiratory symptoms	2 (31)
Skin disorders	2.3 (37)
Sleep disorders	45.3 (720)
Urinary symptoms	22.1 (351)
Other non-motor symptom	10.2 (162)
Missing information	0.8 (13)

**Table 2:** Distribution of non-motor symptoms in idiopathic Parkinson's disease blocking for gender.

	**Females** ** (*n* = 611)**	**Males** ** (*n* = 978)**
**Non-motor symptoms**		
Attention disorders	75 (12.3%)	129 (13.2%)
Cardiovascular symptoms	51 (8.3%)	99 (10.1%)
Cognitive symptoms	100 (16.4%)	166 (17%)
Fatigue	174 (28.5%)	231 (23.6%)
Respiratory symptoms	10 (1.6%)	21 (2.1%)
Gastrointestinal symptoms	157 (25.7%)	219 (22.4%)
Pain	155 (25.4%)	177 (18.1%)
Primary sensory symptoms	57 (9.3%)	68 (7%)
Psychiatric symptoms	317 (51.9%)	367 (37.5%)
Skin disorders	11 (1.8%)	26 (2.7%)
Sleep disorders	271 (44.4%)	449 (45.9%)
Urinary symptoms	123 (20.1%)	228 (23.3%)
Other non-motor symptoms	53 (8.7%)	109 (11.1%)

At least one NMS was present in ≥80% of patients in all H and Y stages, with the highest prevalence (≥90%) documented in H and Y stages 3–5 (see Table 3). For H and Y stages 1 and 2, sleep disorders, followed by psychiatric symptoms, were the most common NMS, whereas for H and Y stages 3–5, the more frequent NMS were psychiatric, followed by sleep disorders. Pain was the third most prevalent complaint in H and Y stages 4–5, whereas fatigue or gastrointestinal problems were the third most frequent symptoms in the other H and Y stages.

**Table 3:** Distribution of non-motor symptoms in idiopathic Parkinson's disease blocking for H and Y score.

	**1 and 1.5** ** (*n* = 85)**	**2** ** (*n* = 813)**	**3** ** (*n* = 431)**	**4 and 5** ** (*n* = 90)**	**Missing score** ** (*n* = 170)**
Patients with at least one NMS	68 (80%)	714 (87.8%)	400 (92.8%)	89 (98.9%)	135 (79.4%)
**Non-motor symptoms**					
Attention disorders	2 (2.4%)	93 (11.4%)	74 (17.2%)	15 (16.7%)	20 (11.8%)
Cardiovascular symptoms	4 (4.7%)	70 (8.6%)	47 (10.9%)	13 (14.4%)	16 (9.4%)
Cognitive symptoms	6 (7.1%)	99 (12.2%)	108 (25.1%)	29 (32.2%)	24 (14.1%)
Fatigue	19 (22.4%)	182 (22.4%)	135 (31.3%)	29 (32.2%)	40 (23.5%)
Gastrointestinal symptoms	9 (10.6%)	198 (24.4%)	120 (27.8%)	30 (33.3%)	19 (11.2%)
Pain	7 (8.2%)	168 (20.7%)	90 (20.9%)	36 (40%)	31 (18.2%)
Primary sensory symptoms	1 (1.2%)	56.0 (6.9%)	48 (11.1%)	8 (8.9%)	12 (7.1%)
Psychiatric symptoms	24 (28.2%)	320 (39.4%)	225 (52.2%)	66 (73.3%)	49 (28.8%)
Respiratory symptoms	0 (0%)	14 (1.7%)	8 (1.9%)	7 (7.8%)	2 (1.2%)
Skin disorders	2 (2.4%)	13 (1.6%)	16 (3.7%)	4 (4.4%)	2 (1.2%)
Sleep disorders	39 (45.9%)	384 (47.2%)	207 (48%)	41 (45.6%)	49 (28.8%)
Urinary symptoms	13 (15.3%)	163 (20%)	107 (24.8%)	33 (36.7%)	35 (20.6%)
Other non-motor symptoms	8 (9.4%)	77 (9.5%)	41 (9.5%)	11 (12.2%)	25 (14.7%)
Missing Information	1 (1.2%)	6 (0.7%)	1 (0.2%)	0 (0%)	5 (2.9%)

NMS were highly prevalent across all age groups, with at least one NMS being reported in more than 85% of patients independently of their age group (see Table 4). Moreover, as shown in Table 5, a high prevalence of NMS (>85%) was reported in all patients with a greater presence in the group of patients who had the disease for a longer period of time (92.1%). Psychiatric disorders and urinary symptoms were more frequent in patients with more years of PD diagnosis. Sleep problems were preeminent in all age groups and across all years after disease diagnosis.

**Table 4:** Distribution of non-motor symptoms in idiopathic Parkinson's Disease blocking for “enrollment age” (percentiles).

	** <62 y.o.** ** <25%** ** (*n* = 368)**	**62–69 y.o.** ** 25–50%** ** (*n* = 376)**	**69–75 y.o.** ** 50–75%(*n =* 392)**	**≥75 y.o.** ** ≥75%** ** (*n* = 453)**
Patients with at least one NMS	317 (86.1%)	335 (89.1%)	351 (89.5%)	403 (89%)
**Non-motor symptoms**				
Attention disorders	41 (11.1%)	40 (10.6%)	62 (15.8%)	61 (13.5%)
Cardiovascular symptoms	20 (5.4%)	32 (8.5%)	41 (10.5%)	57 (12.6%)
Cognitive symptoms	37 (10.1%)	57 (15.2%)	67 (17.1%)	105 (23.2%)
Fatigue	83 (22.6%)	97 (25.8%)	104 (26.5%)	121 (26.7%)
Gastrointestinal symptoms	66 (17.9%)	83 (22.1%)	108 (27.6%)	119 (26.3%)
Pain	73 (19.8%)	85 (22.6%)	84 (21.4%)	90 (19.9%)
Primary sensory symptoms	28 (7.6%)	39 (10.4%)	30 (7.7%)	28 (6.2%)
Psychiatric symptoms	164 (44.6%)	159 (42.3%)	173 (44.1%)	188 (41.5%)
Respiratory symptoms	4 (1.1%)	8 (2.1%)	8 (2%)	11 (2.4%)
Skin disorders	8 (2.2%)	8 (2.1%)	11 (2.8%)	10 (2.2%)
Sleep disorders	162 (44%)	174 (46.3%)	188 (48%)	196 (43.3%)
Urinary symptoms	59 (16%)	84 (22.3%)	98 (25%)	110 (24.3%)
Other non-motor symptoms	38 (10.3%)	47 (12.5%)	34 (8.7%)	43 (9.5%)
Missing information	3 (0.8%)	4 (1.1%)	3 (0.8%)	3 (0.7%)

**Table 5:** Distribution of non-motor symptoms in idiopathic Parkinson's disease blocking for “years from diagnosis” (percentiles).

	** <4 y.** ** <25%** ** (*n* = 338)**	**4–7 y.** ** 25%–50%** ** (*n* = 421)**	**7–11 y. ** **50%–75%** ** (*n* = 411)**	**≥ 11 y.** ** ≥ 75%** ** (*n* = 419)**
Patients with at least one NMS	289 (85.5%)	371 (88.1%)	360 (87.6%)	386 (92.1%)
**Non-motor symptoms**				
Attention disorders	32 (9.5%)	60 (14.3%)	50 (12.2%)	62 (14.8%)
Cardiovascular symptoms	32 (9.5%)	33 (7.8%)	41 (10%)	44 (10.5%)
Cognitive symptoms	45 (13.3%)	65 (15.4%)	69 (16.8%)	87 (20.8%)
Fatigue	74 (21.9%)	120 (28.5%)	92 (22.4%)	119 (28.4%)
Gastrointestinal symptoms	71 (21%)	100 (23.8%)	102 (24.8%)	103 (24.6%)
Pain	66 (19.5%)	85 (20.2%)	78 (19%)	103 (24.6%)
Primary sensory symptoms	20 (5.9%)	24 (5.7%)	34 (8.3%)	47 (11.2%)
Psychiatric symptoms	130 (38.5%)	177 (42%)	174 (42.3%)	203 (48.4%)
Respiratory symptoms	2 (0.6%)	9 (2.1%)	6 (1.5%)	14 (3.3%)
Skin disorders	4 (1.2%)	13 (3.1%)	6 (1.5%)	14 (3.3%)
Sleep disorders	159 (47%)	175 (41.6%)	184 (44.8%)	202 (48.2%)
Urinary symptoms	56 (16.6%)	82 (19.5%)	94 (22.9%)	119 (28.4%)
Other non-motor symptoms	44 (13%)	50 (11.9%)	35 (8.5%)	33 (7.9%)
Missing information	3 (0.9%)	8 (1.9%)	1 (0.2%)	1 (0.2%)

The distribution of NMS in PD for each of the cardinal motor symptoms (rigidity, bradykinesia, tremor, and postural instability) is presented in Supplementary Table 1. Sleep problems and psychiatric disorders were the most frequently observed symptoms independently of the presence of bradykinesia, rigidity, tremor, and postural instability.

## Discussion

In recent years, NMS have assumed an increasingly important role in the clinical characterization of PD due to their frequent and disabling nature, commonly affecting patients' and caregivers' well-being. Although the prevalence of NMS has been described previously (9, 11, 24), there are few studies primarily assessing this prevalence in motor fluctuating PD patients (22). Hence, the aim of the present study was to better recognize the NMS clinical burden in a very large group of motor fluctuating PD patients, obtained from clinical centers located in different European countries and primarily involved in treating PD patients.

The current study documented that almost all patients reported at least one NMS, with psychiatric disorders and sleep problems being the most frequent. The evidence that NMS are significantly present in PD patients was previously confirmed by another large population study (545 patients), carried out in six countries, where the number of concomitant NMS per patient ranged between 9 and 12 (24).

In our population, sleep problems were reported in up to 45% of patients, psychiatric symptoms in up to 43%, fatigue in around 25%, and the frequency of gastrointestinal and urinary symptoms ranged from 22 to 24%. Similarly, previous studies have documented a frequency of depression ranging from 10 to 45% (25–27), anxiety was reported in up to 40% (28–30), sleep problems in 40–98% of PD patients (16, 31, 32), fatigue ranged from 33 to 58% (11, 33, 34) and more than 60% of PD patients complained about urinary symptoms (11, 13, 24, 35).

Although patients with motor-fluctuations were exclusively enrolled, the results were consistent with previous studies including both motor fluctuating and non-fluctuating subjects (10–13). In particular, two large population studies have reported similar results. In the PRIAMO study, a cross-sectional investigation involving 1,072 PD patients at different motor stages, psychiatric symptoms were the most frequently reported (66.8%), followed by sleep disorders (64.1%), gastrointestinal symptoms and pain (61.0%), fatigue (58.1%), urinary symptoms (57.3%), and attention/memory deficits (44.7%) (11). In another multinational study, performed by Martinez-Martin et al., urinary symptoms were the most frequent (59%) followed by depression/anxiety (48%), attention/memory deficit (42%), and sleep problems (37%) (24).

Although our findings are substantially consistent with these previous studies, when we consider the frequency of the single individual symptoms some differences in prevalence could be observed. For instance, when compared with findings from previous studies, the frequency of pain and gastrointestinal and urinary symptoms were lower in the present study. This may be explained by some discrepancies in methodological aspects, including recruitment criteria and tools used to assess NMS. Consistently, previous studies assessed the NMS through a specific questionnaire, whereas in the current investigation no validated instrument was used since the NMS were collected by history and clinically based interviews.

Since gender-related differences in brain structure and function, as well as age-related physical changes, may lead to differences in the clinical expression of diseases, it is important to identify the spectrum and severity of NMS for males and females in order to provide a treatment targeting gender-based needs. In the present study, psychiatric symptoms were highly frequent among women, while the most common NMS among men was sleep disorders. Martinez-Martin et al. (36) have reported, in part, similar results. In particular, they found that women showed a higher frequency in several NMS, including depression/anxiety, cardiovascular symptoms, and the miscellaneous domain, while sexual–function-related symptoms prevailed in men.

Among the NMS in motor-fluctuating PD patients, in previous studies sleep problems were reported in 45–47% of patients at H and Y stages 1 and 2, representing the most common NMS in the early stages of the disease, followed by psychiatric disorders with a prevalence of 28% in stage 1 and 39% in stage 2. Conversely, evidence showed a high prevalence of psychiatric NMS, mainly depression, in the initial or even prodromal phase of PD (11). However, in the present study, psychiatric disorders (also counting some behavioral disturbances typical of the advanced stages of the disease, such as hallucinations and delirium) were the most frequent symptoms for the more severe H and Y stages 3–5, followed by sleep disorders. Furthermore, pain frequency and urinary symptoms increased with disease severity, whereas fatigue or gastrointestinal problems were more common symptoms across all disease stages. In particular, urinary symptoms were frequently reported (36.7%) at H and Y stages 4–5, which is consistent with previous studies (11, 24). This may in part be related to the natural onset of urinary disorders with increasing age.

These findings are in line overall with the observed relation between NMS and PD severity, with an increase of NMS with the progression and duration of the disease (11). Hence, these results suggest that NMS should target medical interventions in PD patients not only in the later stages of the disease but also in the earliest PD motor fluctuations.

The current study has some limitations that need to be addressed. First, no healthy controls or active controls (e.g., patients without motor fluctuation) were considered in order to assess the differences that exist when they are compared with PD patients. Second, the assessment of NMS was collected only at baseline from amnestic data and clinically based interviews, and thus, the lack of a validated disease-specific measure may have underestimated the frequency of some NMS. Third, since the study was conducted on patients who could receive safinamide, the majority of the patients were in the H and Y stages 2 and 3. Therefore, the frequency of NMS should be generalized with caution, especially in patients in advanced stages of the disease. Fourth, all the PD patients included were treated with dopamine agonists and/or levodopa, and the possible mediated effects of these drugs on NMS cannot be evaluated. Fifth, no data on higher brain function were evaluated. Although no differences among countries were detected, each country may choose different drug options (e.g., deciding not to use safinamide), which may influence the NMS prevalence and burden. Finally, the design of the study did not allow the assessment of non-motor fluctuations, and future studies are needed to assess the percentage of fluctuating NMS since they can be a determinant of quality of life in PD patients (37). Nonetheless, these limitations were counterbalanced by the large sample size of PD patients with motor fluctuations included across all stages and from different European outpatient clinics.

In conclusion, differences in the clinical manifestation of NMS by PD patients may have important implications in management and prognosis. The findings of the present multinational study reinforce the increasing need to also consider NMS in clinical practice and take into account their different prevalence across the stages of the disease, the influence of gender and the clinical PD characteristics.

## Data Availability Statement

Publicly available datasets were analyzed in this study. The final report of the study is publicly available on the EUPAS Register with the following accession number: EUPAS 13745.

## Ethics Statement

The studies involving human participants were reviewed and approved by the Independent Ethics Committees and Health Authorities in each country involved in the study and was conducted according to the ethical standards of the institutional and/or national research committee and the Declaration of Helsinki. The patients/participants provided their written informed consent to participate in this study.

## Author Contributions

CL and MF prepared the manuscript. EB was responsible for the statistical analysis. RC handled the data collection for this analysis. NBM, MP, and AS revised the manuscript and prepared a critical revision of it. CC revised the manuscript and supported the preparation of the manuscript. All authors approved the final version of the manuscript.

## Conflict of Interest

CC is a Zambon S.p.A. employee; EB is a statistical consultant of Zambon S.p.A.; CL and AS received symposium honoraria from Zambon S.p.A. The remaining authors declare that the research was conducted in the absence of any commercial or financial relationships that could be construed as a potential conflict of interest.
