# Supplementary material for: Frequency of Non-motor Symptoms in Parkinson's Patients With Motor Fluctuations
Source: Front Neurol. 2021 Jun 29;12:678373. doi: 10.3389/fneur.2021.678373 (PMC8276257; doi:10.3389/fneur.2021.678373)
Supplement: Supplementary file 1 [file Table_1.DOCX]

Supplementary Material

## Supplemental Table 1. Distribution of Non-motor symptoms in Idiopathic Parkinson’s Disease blocking for Motor symptoms

|  | **Bradykinesia** | | **Rigidity** | | **Tremor** | | **Postural Instability** | |
| --- | --- | --- | --- | --- | --- | --- | --- | --- |
| **Non motor symptom**  ***% (n)*** | **Yes**  **(n=1396)** | **No**  **(n=193)** | **Yes**  **(n=1310)** | **No**  **(n=279)** | **Yes**  **(n=917)** | **No**  **(n=672)** | **Yes**  **(n=482)** | **No**  **(n=1107)** |
| Attention disorders | 14 (195) | 4.7 (9) | 13.8 (181) | 8.2 (23) | 14.1 (129) | 11.2 (75) | 19.1 (92) | 10.1 (112) |
| Cardiovascular symptoms | 9.4 (131) | 9.8 (19) | 9.2 (121) | 10.4 (29) | 9.8 (90) | 8.9 (60) | 12.2 (59) | 8.2 (91) |
| Cognitive symptoms | 17.8 (249) | 8.8 (17) | 17.6 (230) | 12.9 (36) | 16.9 (155) | 16.5 (111) | 26.3 (127) | 12.6 (139) |
| Fatigue | 27.4 (382) | 11.9 (23) | 26.5 (347) | 20.8 (58) | 27.3 (250) | 23.1 (155) | 31.3 (151) | 22.9 (254) |
| Respiratory symptoms | 1.9 (27) | 2.1 (4) | 1.6 (21) | 3.6 (10) | 2.2 (20) | 1.6 (11) | 3.5 (17) | 1.3 (14) |
| Gastrointestinal symptoms | 24.0 (335) | 21.2 (41) | 24 (314) | 22.2 (62) | 25.6 (235) | 21 (141) | 28.6 (138) | 21.5 (238) |
| Pain | 21.9 (306) | 13.5 (26) | 22.1 (290) | 15.1 (42) | 22.4 (205) | 18.9 (127) | 27.4 (132) | 18.1 (200) |
| Primary sensory symptoms | 8.4 (117) | 4.1 (8) | 8.7 (114) | 3.9 (11) | 9.2 (84) | 6.1 (41) | 10.6 (51) | 6.7 (74) |
| Psychiatric symptoms | 44.6 (623) | 31.6 (61) | 44.5 (583) | 36.2 (101) | 43.9 (403) | 41.8 (281) | 55 (265) | 37.9 (419) |
| Skin disorders | 2.6 (36) | 0.5 (1) | 2.5 (33) | 1.4 (4) | 2.8 (26) | 1.6 (11) | 3.5 (17) | 1.8 (20) |
| Sleep disorders | 47.2 (659) | 31.6 (61) | 46.1 (604) | 41.6 (116) | 46.7 (428) | 43.5 (292) | 48.8 (235) | 43.8 (485) |
| Urinary symptoms | 23.2 (324) | 14 (27) | 21.8 (286) | 23.3 (65) | 23.2 (213) | 20.5 (138) | 27.4 (132) | 19.8 (219) |
| Other nonmotor symptom | 10.3 (144) | 9.3 (18) | 9.8 (129) | 11.8 (33) | 10.8 (99) | 9.4 (63) | 10.6 (51) | 10 (111) |
